# Pulmonary Rehabilitation With Balance Training for Fall Reduction in Chronic Obstructive Pulmonary Disease: Protocol for a Randomized Controlled Trial

**DOI:** 10.2196/resprot.8178

**Published:** 2017-11-20

**Authors:** Marla K Beauchamp, Dina Brooks, Cindy Ellerton, Annemarie Lee, Jennifer Alison, Pat G Camp, Gail Dechman, Kimberley Haines, Samantha L Harrison, Anne E Holland, Alda Marques, Rahim Moineddin, Elizabeth H Skinner, Lissa Spencer, Michael K Stickland, Feng Xie, Roger S Goldstein

**Affiliations:** ^1^ School of Rehabilitation Science McMaster University Hamilton, ON Canada; ^2^ Department of Respiratory Medicine West Park Healthcare Centre Toronto, ON Canada; ^3^ Department of Physical Therapy University of Toronto Toronto, ON Canada; ^4^ Department of Rehabilitation, Nutrition and Sport LaTrobe University Melbourne Australia; ^5^ Institute for Breathing and Sleep Melbourne Australia; ^6^ Department of Physiotherapy School of Primary and Allied Health Care Monash University Melbourne Australia; ^7^ Faculty of Health Sciences University of Sydney Lidcombe Australia; ^8^ Sydney Local Health District Camperdown Australia; ^9^ Department of Physical Therapy University of British Columbia Vancouver, BC Canada; ^10^ Centre for Heart Lung Innovation University of British Columbia Vancouver, BC Canada; ^11^ School of Physiotherapy Dalhousie University Halifax, NS Canada; ^12^ Department of Physiotherapy Western Health Melbourne Australia; ^13^ School of Health and Social Care Teesside University Middlesbrough United Kingdom; ^14^ Alfred Health Melbourne Australia; ^15^ Respiratory Research and Rehabilitation Laboratory School of Health Sciences University of Aveiro Aveiro Portugal; ^16^ Institute for Biomedicine Research University of Aveiro Aveiro Portugal; ^17^ Department of Family and Community Medicine University of Toronto Toronto, ON Canada; ^18^ Faculty of Medicine, Nursing and Health Sciences Monash University Melbourne Australia; ^19^ Division of Pulmonary Medicine Department of Medicine University of Alberta Edmonton, AB Canada; ^20^ GF MacDonald Centre for Lung Health Edmonton, AB Canada; ^21^ Department of Health Research Methods, Evidence, and Impact McMaster University Hamilton, ON Canada; ^22^ Research Institute of St. Joseph’s Hamilton, ON Canada; ^23^ Program for Health Economics and Outcome Measures Hamilton, ON Canada; ^24^ Department of Medicine University of Toronto Toronto, ON Canada

**Keywords:** COPD, pulmonary rehabilitation, balance, exercise, falls, economic analysis

## Abstract

**Background:**

Chronic obstructive pulmonary disease (COPD) is a leading cause of morbidity and mortality worldwide. A growing body of evidence shows that individuals with COPD have important deficits in balance control that may be associated with an increased risk of falls. Pulmonary rehabilitation (PR) is a key therapeutic intervention for individuals with COPD; however, current international guidelines do not include balance training and fall prevention strategies.

**Objective:**

The primary aim of this trial is to determine the effects of PR with balance training compared to PR with no balance training on the 12-month rate of falls in individuals with COPD. Secondary aims are to determine the effects of the intervention on balance, balance confidence, and functional lower body strength, and to estimate the cost-effectiveness of the program.

**Methods:**

A total of 400 individuals from nine PR centers across Canada, Europe, and Australia will be recruited to participate in a randomized controlled trial. Individuals with COPD who have a self-reported decline in balance, a fall in the last 2 years, or recent near fall will be randomly assigned to an intervention or control group. The intervention group will undergo tailored balance training in addition to PR and will receive a personalized home-based balance program. The control group will receive usual PR and a home program that does not include balance training. All participants will receive monthly phone calls to provide support and collect health care utilization and loss of productivity data. Both groups will receive home visits at 3, 6, and 9 months to ensure proper technique and progression of home exercise programs. The primary outcome will be incidence of falls at 12-month follow-up. Falls will be measured using a standardized definition and recorded using monthly self-report fall diary calendars. Participants will be asked to record falls and time spent performing their home exercise program on the fall diary calendars. Completed calendars will be returned to the research centers in prepaid envelopes each month. Secondary measures collected by a blinded assessor at baseline (pre-PR), post-PR, and 12-month follow-up will include clinical measures of balance, balance confidence, functional lower body strength, and health status. The cost-effectiveness of the intervention group compared with the control group will be evaluated using the incremental cost per number of falls averted and the incremental cost per quality-adjusted life years gained.

**Results:**

Recruitment for the study began in January 2017 and is anticipated to be complete by December 2019. Results are expected to be available in 2020.

**Conclusions:**

Findings from this study will improve our understanding of the effectiveness and resource uses of tailored balance training for reducing falls in individuals with COPD. If effective, the intervention represents an opportunity to inform international guidelines and health policy for PR in individuals with COPD who are at risk of falling.

**Trial Registration:**

ClinicalTrials.gov NCT02995681; https://clinicaltrials.gov/ct2/show/NCT02995681 (Archived by WebCite at http://www.webcitation.org/6ukhxgAsg)

## Introduction

Chronic obstructive pulmonary disease (COPD) is a major cause of morbidity and mortality and is expected to be among the top three causes of death in the world by the year 2020 [1]. As the population ages, the prevalence of COPD is expected to increase, which will impact health care resource utilization [1]. In 2010, the Centers for Disease Control and Prevention reported the medical costs attributable to having COPD were US $32.1 billion and by the year 2020, this is expected to increase to US $49 billion [2].

Although treatment of COPD is often focused on respiratory function, secondary impairments in skeletal muscle function, mobility, and exercise capacity are well recognized [3-5]. There is now strong evidence that individuals with COPD also have important deficits in balance control and an increased risk of falls [6-17], which has been linked to increased morbidity and mortality in this population [18,19].

Balance impairment is widely recognized as one of the most important modifiable risk factors for falls in older adults [20]. Numerous studies have documented impairments in clinical and laboratory measures of balance in individuals with varying COPD severity compared to controls [6,11,12,14-17,21,22]. Underlying mechanisms for reduced balance in COPD may include decreased levels of physical activity [11,12], peripheral muscle weakness [11], altered trunk muscle mechanics [16], hypoxemia [23], and somatosensory deficits [24]. Our work has also shown that individuals with COPD have a unique profile of balance impairment, with marked deficits in three specific subsystems of balance: biomechanics (ie, strength, range of motion, and posture), transitions (ie, change in body positions), and gait (ie, stability while walking) [17].

There is growing recognition of an increased fall risk in people with COPD [7-10]. In addition to impaired balance, other fall risk factors in COPD include the use of multiple medications, muscle weakness, cognitive impairment, and comorbidities such as osteoarthritis and osteoporosis [7]. COPD was second only to osteoarthritis in its association with the number of falls in a large cohort study of elderly women [9] and was the only chronic condition that predicted falls in a further cohort study of 16,000 participants [8]. Patients with COPD have reported a higher prevalence of falls (40%-55%) [10,11,15,17] than the general elderly population and in two prospective studies [7,10], the annual fall rate in COPD was up to five times higher than expected based on age alone. Fall-related injuries as well as associated hospitalizations are also common in people with COPD [18,25]. In those with more severe disease and depression, a history of falling is a strong predictor of mortality [19]. These data emphasize the need for the development of fall prevention strategies specific to individuals with COPD.

Pulmonary rehabilitation (PR) is recommended as standard management for COPD as there is strong evidence for its effect on improving symptoms, exercise tolerance, and health-related quality of life [26-29]. The program typically consists of supervised exercise training, disease-specific education, self-management, and psychosocial support. While exercise is viewed as the cornerstone of PR, it is largely focused on aerobic exercise to increase endurance and resistance training. International guidelines for PR do not include balance training nor fall prevention strategies and few programs include a balance assessment [26-31]. We have shown that the exercise component of conventional PR has only minimal effect on measures of balance and fall risk [32]. Therefore, to reduce falls, exercise that includes targeted balance training is needed [33]. We recently undertook a pilot randomized controlled trial (RCT) [34] to examine the effects of adding balance training alongside PR, which specifically addressed the unique profile of balance deficits we had identified in COPD [17]. We reported large and clinically important improvements in balance performance among those in the intervention group compared to controls who completed typical PR [34]. We have also found such an intervention to be easily implementable into clinical practice [35]. This study builds on our existing experience to rigorously test the effect of the intervention on balance and fall reduction in individuals with COPD.

The objectives of this study are to (1) evaluate the long-term effects of a tailored, balance exercise program on the 12-month rate of falls in individuals with COPD who are enrolled in outpatient PR compared to PR with balance exercise training; (2) determine the effects of a tailored, balance exercise program on the secondary outcomes, including measures of balance, balance confidence, and functional lower body strength; and (3) conduct an economic analysis to evaluate the cost-effectiveness of the program compared to PR with no balance training.

## Methods

### Ethics

The protocol has received ethics approval from the Joint West Park Healthcare Centre/Toronto Central Community Care Access Centre/Toronto Grace Health Centre Research Ethics Board (REB) (Canada); the University of Toronto REB (Canada); the Nova Scotia Health Authority REB (Canada); the University of Aveiro REB (Portugal); the University of British Columbia/Providence Health Care REB (Canada); the National Health Service REB (United Kingdom); the University of Alberta REB (Canada); the Alfred Health REB (Australia); the Royal Prince Alfred Hospital REB (Australia); and the Western Health REB (Australia).

Adverse events will be collected after individuals have consented and been enrolled in the study. Adverse events that meet the criteria for a serious adverse event (SAE) will be reported to the local REB. An SAE is defined as any adverse event that results in the following patient outcomes: death, life-threatening condition, hospitalization (initial/ prolonged), disability/ permanent damage, intervention required to prevent permanent impairment or damage, or any other medically important event [36].

Formal amendments will be submitted to each REB in consultation with local investigators should there be any modifications to the protocol. Modifications to the protocol will be final and agreed upon by all investigators before amendment applications are submitted.

### Trial Registration and Reporting

The trial is registered with ClinicalTrials.gov (NCT02995681) and our protocol is reported according to the Standard Protocol Items: Recommendation for Intervention Trials (SPIRIT) checklist [37].

### Study Design

**Figure 1:**
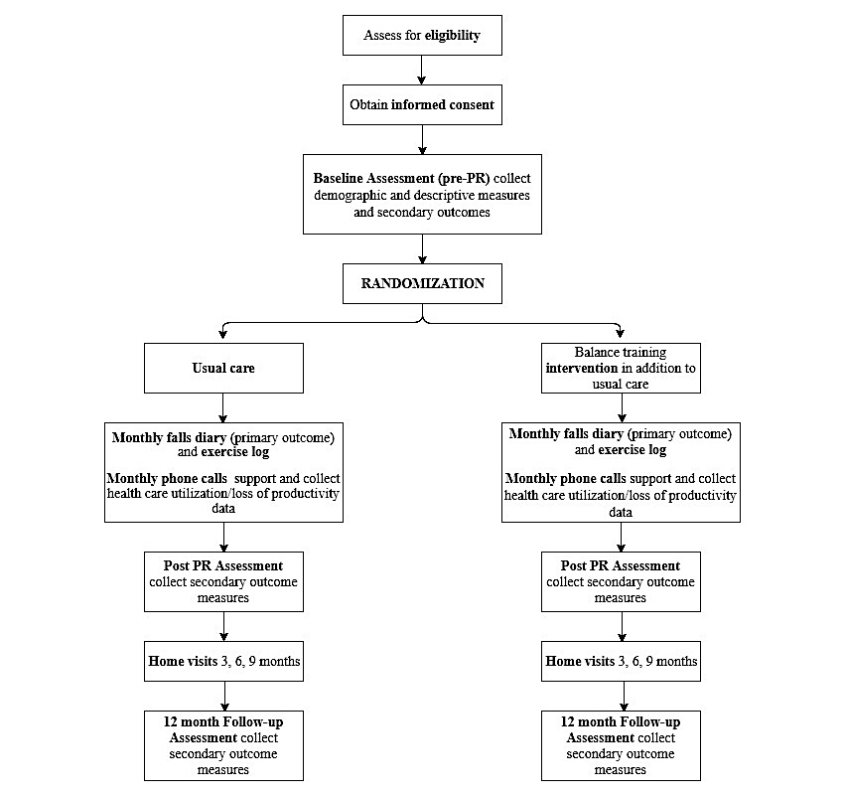
Participant flow diagram. PR: pulmonary rehabilitation.

We will conduct a multi-center RCT with allocation concealment as well as blinding of the outcome assessors and data analysts to group allocation. A participant flowchart is outlined in Figure 1.

### Setting

Outpatient PR programs at nine sites across four countries—Canada, United Kingdom, Portugal, and Australia—are participating in the study. PR programs in Canada, United Kingdom, and Australia are publicly funded through government-run health plans. PR programs in Portugal are only partially funded by the government.

### Participants

Individuals will be considered eligible for the study if they meet the following inclusion criteria: (1) have a diagnosis of COPD based on the Global Initiative for Chronic Obstructive Lung Disease (GOLD) criteria—postbronchodilator forced expiratory volume in 1 second (FEV_1_)/forced vital capacity (FVC) ratio <70% [38]; (2) have a self-reported decline in balance, a fall in the last 2 years, or a recent near fall; and (3) are able to provide written informed consent.

Individuals will be excluded if they meet the following exclusion criteria: (1) are unable to communicate because of language skills (eg, communication deficit; non-English speaking in Canada, United Kingdom, or Australia; or non-Portuguese speaking in Portugal); (2) are hearing or cognitively impaired (eg, dementia or neurological condition); or (3) have evidence of a neurological or musculoskeletal condition that severely limits mobility and postural control (eg, cerebrovascular accident, Parkinson’s disease, or lower limb amputee).

### Recruitment

Individuals will be recruited upon enrolment in PR programs at each participating site. Study information sheets were developed specific to each site and translated as required. A member of the research team not involved in the individual’s clinical care will meet with potential participants to provide information about the study. If the individual agrees to participate, written informed consent will be obtained.

Recruitment will continue until the overall target population size is reached for the study. Each clinical site involved in the trial was selected based on investigator expertise and data on previous experience with study recruitment rates.

### Retention

Participants may withdraw from the study for any reason at any time. Investigators may also withdraw participants from the study if they demonstrate a sudden and severe deterioration in balance (eg, due to dizziness from a medication change or a newly diagnosed medical condition) that would jeopardize their safety during balance training.

Every effort will be made to perform study assessments on individuals who withdraw from the study or who do not sufficiently adhere to the usual care or intervention protocols.

### Allocation

A computer-generated block randomization table will be created by a member of the research team not involved in recruitment. A randomization schedule will be provided for each site. After an individual has been enrolled in the study and the baseline assessment has been completed, the central research coordinator will consecutively allocate participants using sealed opaque envelopes and advise each site coordinator of group allocation.

### Intervention

#### Control: Usual Care

Participants randomized to the control group will receive usual outpatient PR. The PR programs across the nine sites are given two to three times per week for a total duration of 8-12 weeks depending on the site. All PR programs are offered in accordance with international guidelines [26-29,31] and consist of all core components of rehabilitation including aerobic and resistance exercise, self-management education, and psychosocial support.

Upon discharge from outpatient PR, participants will receive the usual individualized home-based exercise program which will include walking and lower extremity resistance exercises two to three times a week. To control for attention between the two groups and maximize data collection, in addition to usual care, participants will receive monthly phone calls from a physiotherapist to provide support and problem solve any issues, as well as home visits at 3, 6, and 9 months.

#### Intervention: Tailored Balance Training

Participants randomized to the intervention group will receive a 30-minute balance training session twice per week in addition to usual PR, and will be asked to complete a third session at home each week, for a total of 90 minutes of balance training per week. This is in keeping with best practice guidelines for older adults and the design of our previous RCT in COPD [32,35,39]. The balance exercises (see Multimedia Appendix 1) will be tailored to emphasize the areas found to be most deficient in COPD [17]. Participants will receive balance training in a separate location or at a separate time from the control group. Sessions will be supervised by physiotherapists using a circuit training approach. While participants may work through stations in groups, each participant will receive individualized exercise prescription based on their baseline balance assessment, as well as recommendations for individualized exercise progression. Our previous trial demonstrated the feasibility of this approach [34]. Participants will also receive an instruction booklet that outlines their individual exercises for the once-weekly, home-based balance training session during PR.

After completion of PR, participants will complete the prescribed home-based balance training program three times per week. The home-based program will follow for the duration of the 12-month follow-up period.

Participants will receive monthly phone calls from their physiotherapist to provide support and problem solve any issues, as well as home visits at 3, 6, and 9 months to ensure proper technique and progression at home.

### Outcomes

Outcome measures will be collected by a blinded assessor at baseline, at the end of PR, and at 12-month follow-up.

#### Primary Outcome

The primary outcome measure will be incidence of falls at 12-month follow-up. A fall will be defined as “an incident in which the body unintentionally comes to rest on the ground or other lower level which is not as a result of a violent blow, loss of consciousness, sudden onset of paralysis as in a stroke or an epileptic seizure” [40]. Based on published consensus [41], the participants will be asked, “Have you had any fall including a slip or trip in which you lost your balance and landed on the floor or ground or lower level?” Falls will be measured using monthly fall diary calendars, which are the recommended methodology for falls reporting in clinical trials [41]. All participants will receive the fall diary calendars at the time of the baseline assessment.

Participants will be asked to record falls and time spent performing their home exercise program on the fall diary calendars. They will also be asked to contact the research assistant in the event of a fall or health-related event to ensure all relevant information is included and to return completed diaries in prepaid envelopes to the research center each month. Participants who do not return the calendars or those with unclear data will be telephoned to collect the information.

#### Secondary Outcomes

##### Clinical Measures of Balance

The Berg Balance Scale (BBS) [42] is one of the most widely used and psychometrically robust clinical measures of balance for older adults. It assesses 14 tasks such as transfers, reaching, turning around, and single-legged stance. Evidence supports the BBS’s construct validity and sensitivity to change following PR in individuals with COPD [43]. Test-retest reliability and predictive validity for fall risk have been demonstrated in community-dwelling older adults [44]. The total score is out of 56 with higher scores indicating better balance. Among patients with moderate to severe COPD undergoing PR, a change of 5-7 points is clinically important [45].

The Balance Evaluation Systems Test (BESTest) [46] is a comprehensive 36-item balance evaluation tool that assesses six subsystems of balance control: biomechanics, stability limits/verticality, anticipatory postural adjustments for postural transitions, reactive postural response strategies, weighting of sensory information, and postural stability during gait. Evidence supports the BESTest’s construct validity in COPD [43] and sensitivity to change, as well as test-retest reliability and concurrent validity in adults with and without balance disorders [46]. Total scores range from 0% to 100% and a change of 13-17 points on the BESTest is clinically important in patients with COPD [45].

##### Balance Confidence

The Activities-Specific Balance Confidence (ABC) scale [47] requires patients to indicate their confidence in performing 16 activities without losing their balance or becoming unsteady. The ABC scale has good test-retest reliability and predicts falls in older adults residing in the community [41,44]. In patients with COPD, the ABC scale has demonstrated construct validity as well as criterion validity for falls [43]. Total scores range from 0% to 100% with higher scores for better balance confidence. A change of 19% reflects a clinically important difference for this measure in COPD [45].

##### Functional Lower Body Strength

The 30-second repeated chair stand test [48] will be used as a measure of functional lower body strength. The reliability and validity of this measure has been established in people with COPD and the measure is correlated to maximal voluntary force from a seated leg press [49]. The repeated chair stand test is sensitive to change after balance training in people with COPD [34].

##### Descriptive Measures

Descriptive measures will include sociodemographic variables (gender, age), anthropometry (height, weight, body mass index), smoking history, oxygen use, rollator walker use, spirometry measures (FEV_1_, FVC, FEV_1_/FVC ratio, FEV_1_% predicted, FVC % predicted), a measure of exercise tolerance (6-minute walk test [6MWT]) [50], comorbid conditions, and medication use. We will also use the Baseline Dyspnea Index (BDI) [51] and the Falls Risk for Older People-Community setting (FROP-Com) [52] to further characterize the study sample. A home exercise log will be used to describe participant self-reported adherence to the intervention.

##### Health Status

Health status is an important patient-reported outcome that describes health-related quality of life of patients. The European Quality of Life 5-Dimension Questionnaire (EQ-5D-5L) will be used to measure health status. It consists of five questions covering dimensions of mobility, self-care, usual activities, pain/discomfort, and anxiety/depression with five response options indicating *no*, *mild*, *moderate*, *severe*, and *extreme* problems for each dimension [53]. The answers to the five questions included in this instrument can be converted to health utility which is an index score anchored at 0 (equivalent to dead) and 1 (for full health), with negative scores for worse than dead. The EQ-5D-5L-derived health utilities will be calculated using the recently developed scoring algorithm for Canada [54] and United Kingdom [55] and the interim algorithm for Australia and Portugal [56]. The EQ-5D-5L will be administered at baseline and at 6- and 12-month follow-up, and then used to determine quality-adjusted life years (QALY) for each respondent. For this study, the resulting adjustment is an adjustment on quality of life as we do not anticipate any differences in survival.

### Economic Analysis

An economic analysis will be conducted alongside the trial using the primary outcome (ie, number of falls) and health utilities as effect measures. We will use methods consistent with standard texts and guidelines for economic evaluation [57]. The time horizon of the analysis will be the period of study follow-up (ie, 12 months). Both societal and health care payers’ perspectives will be used for the analysis.

Health care resource utilization and loss of productivity related to falls and COPD will be collected during monthly phone calls to participants. During the monthly phone calls, the participants will be asked if they have had any health care appointments (ie, family doctor, walk-in/urgent care clinic, or specialist), emergency room visits, or hospital stays related to having COPD or a fall. If the participant responds “yes,” the physiotherapist will then ask for details about which health care professionals were seen; what tests, medications, or treatments were ordered; and, if they were hospitalized, for how long.

The time it takes for therapists to administer the rehabilitation exercises, monthly phone calls, and home visits in both intervention and control groups will also be recorded. These resource utilizations will be converted to direct medical costs using country-specific unit costs. Indirect costs may include the productivity loss for patients, if still working, or family members who provide care/assistance to the participating patient.

### Data Collection

All subject data will be deidentified and coded with a unique study identifier. Data collection forms and digital files will be securely stored as per the REB protocols.

Each site will be responsible for entering deidentified participant data into a digital database and securely sharing it with the lead investigators and central data analyst for analysis. Only the lead investigators and central data analyst will have access to the final dataset.

A data monitoring committee will be set up to monitor the trial for possible harmful effects. The committee will evaluate any adverse events to recommend whether the study should continue, be modified, or stopped due to safety concerns.

### Data Analysis

The data analysts on the research team will be blinded to group allocation. Descriptive summary statistics will be reported using mean, median, and standard deviation for continuous measures, and frequency and counts for categorical variables. For the primary outcome, we will use random effect Poisson (Negative Binomial) regression model to compare the number of falls at 12-month follow-up between intervention and control groups adjusting for potential confounders, repeated measures, and clustering of participants within centers. For secondary outcomes, we will use random effect linear regression to compare intervention and control groups adjusting for potential confounders and clustering of the observations. For all measures, we will perform intention-to-treat and per protocol analysis.

We will estimate the total cost for the intervention and control groups and calculate the difference between the two groups. Similarly, for the effect measures, we will calculate the difference in the number of falls and in the QALY between groups. The summary measure for the economic analysis will be the incremental cost per number of falls averted (cost-effectiveness analysis) and the incremental cost per QALY gained (cost-utility analysis) for the intervention group compared with the control. Given that this economic analysis will be using data primarily from study participants, nonparametric bootstrap will be used to calculate the 95% confidence interval around the incremental ratios. The decision uncertainty will be presented using cost-effectiveness acceptability curves that show the probability of the intervention being cost-effective compared with the control group at a wide range of willingness to pay for a QALY [57].

No interim or subgroup analysis is planned. We will perform both intention-to-treat and per protocol analyses.

### Sample Size Calculation

First, we calculated the sample size for an individually randomized two-arm study with 80% power and 5% type I error. We estimated the rate of falls for the control group as 120 per 100-person years, consistent with prospective studies documenting fall rates in similar populations of patients with COPD in Canada and Australia [7,10]; the rate of falls in the intervention group was estimated as 84 per 100-person years, which corresponds to a 30% reduction in the rate of falls. A 30% reduction in fall rate is widely used as a clinically significant threshold in fall prevention studies and is consistent with meta-analyses demonstrating the effect of multi-component exercise on fall rate. Based on this data, the estimated number of participants in each arm with 1-year follow up is 124 patients. However, our study is a multicenter study in which participants are clustered within nine centers. Assuming a coefficient of variation of size 0.10, we need, on average, 19 participants in each arm per site, corresponding to a total of 171 participants in each arm, to account for the clustering of the study [58].

Assuming a similar clustering effect for the secondary outcomes with a sample size of 171 participants in each arm and nine centers, our study has 80% power with 5% type I error to detect differences of the following magnitudes (MCID: minimal clinically important difference):

1. Berg Balance (SD=6.15, MCID=5) differences of 3.0 units or larger.
2. BESTest (SD=13, MCID=13) differences of 6.4 units or larger.
3. ABC (SD=23.2, MCID=19) differences of 11.5 units or larger.
4. Chair stand (SD=4.8, MCID=3) differences of 2.4 units or larger.

These values are close to half the standard deviation of the outcome measures and are sufficient to detect clinically important changes [45].

We anticipate a loss to follow-up of 15%-20% based on previous trials in PR and have inflated our sample size to 400 participants (200 in each arm) to account for this. Should a participant be unable to attend an assessment session at a study center, we will minimize loss to follow-up by offering to complete assessments in the participants’ homes.

## Results

A Canadian Institutes of Health Research (CIHR) project grant was received to fund the study. Recruitment began in January 2017 and is anticipated to be completed by December 2019. Results are expected to be available in 2020.

## Discussion

### Principal Findings

The high incidence of falls and balance problems in people with COPD underscores the need for evidence-based approaches to fall prevention in this population. While studies have shown that balance training alongside PR improves measures of balance and mobility, the long-term impact on falls has not been determined. This international multi-center trial will be the first RCT of tailored balance training in people with COPD who are enrolled in outpatient PR. It is also the first study of balance training that includes an economic analysis to consider the cost-effectiveness of the intervention.

If participation in tailored balance training does decrease falls compared to usual PR, this approach will represent an innovative and potentially cost-saving strategy to prevent falls and reduce health care utilization in COPD. Our results will be relevant for guiding clinical and policy-based decision-making, given the high prevalence of COPD and potential for severe consequences of falls in this population.

### Conclusions

In conclusion, this study will provide evidence on the effectiveness of tailored balance training for individuals with COPD who have a history of falling or a self-reported decline in balance skills. If effective, the results of this study could be used to inform future international guidelines and health policy for PR in this population.

## Appendix

Multimedia Appendix 1 Balance training exercise flow sheet.

Multimedia Appendix 2 CIHR peer-review report.

## Abbreviations

6MWT: 6-minute walk test
ABC: Activities-Specific Balance Confidence
BBS: Berg Balance Scale
BDI: Baseline Dyspnea Index
BESTest: Balance Evaluation Systems Test
CIHR: Canadian Institutes of Health Research
COPD: chronic obstructive pulmonary disease
EQ-5D-5L: European Quality of Life 5-Dimension Questionnaire
FEV1: forced expiratory volume in 1 second
FROP-Com: Falls Risk for Older People-Community setting
FVC: forced vital capacity
GOLD: Global Initiative for Chronic Obstructive Lung Disease
MCID: minimal clinically important difference
PR: pulmonary rehabilitation
QALY: quality-adjusted life years
RCT: randomized controlled trial
REB: Research Ethics Board
SAE: serious adverse event
SPIRIT: Standard Protocol Items: Recommendation for Intervention Trials
