# Supplementary material for: Pulmonary Rehabilitation With Balance Training for Fall Reduction in Chronic Obstructive Pulmonary Disease: Protocol for a Randomized Controlled Trial
Source: JMIR Res Protoc. 2017 Nov 20;6(11):e228. doi: 10.2196/resprot.8178 (PMC5715200; doi:10.2196/resprot.8178)
Supplement: Multimedia Appendix 1 [file resprot_v6i11e228_app1.pdf]

Subject Name: ______________________________ **Start Date:** ___________________________

Date (mm/dd)								
**Healthcare Practitioner Initials**								

STANCE EXERCISES

1. Narrow Stance (NS)

Exercise Session	1	2	3	4	5	6	7	8
NS – w/out support (1min)								
NS - eyes closed (30 sec)								
NS - reaching beyond base of support								
NS - throw and catch ball								
NS - perturbations (forward, back, sideways)								
NS- ramp								

2. Tandem Stance (TS)

Exercise Session	1	2	3	4	5	6	7	8
TS – w/out support (30 sec)								
TS - reaching beyond base of support								
TS - throw and catch ball								
TS - eyes closed (20 sec)								
TS - perturbations (forward, back, sideways)								

3. One-Legged Stance (OLS) (with light upper extremity support if needed e.g., back of chair or light touch)

Exercise Session	1	2	3	4	5	6	7	8
OLS - (30 sec)								
OLS - spell names with foot								
OLS - leg out to side (10 sec hold)								
OLS - throw and catch ball								
OLS - eyes closed (20 sec)								

✓= successful, ✻ = Try Again, P = Pain, FT = fingertip, D= Dizzy

Modified from: Beauchamp M, Janaudis-Ferreira T, Parreira V et al. A randomized controlled trial of balance training during pulmonary rehabilitation for individuals with COPD. Chest 2013;144:1803-10.

4. Stand on Uneven Surfaces

Exercise Session	1	2	3	4	5	6	7	8
Foam - normal stance (30 sec)								
Foam - narrow stance, eyes open (1min)								
Foam - narrow stance, eyes closed (1min)								
Foam - normal stance, reaching beyond BOS								
Foam - normal stance, catch ball								
Wobble board - record max time								
Wobble board - dance/arm movements								
Bosu ball - record max time								
Bosu ball - catch ball								

TRANSITION EXERCISES

1. Sit to Stand (STS)

Exercise Session	1	2	3	4	5	6	7	8
STS - high chair using arms (5 reps)								
STS - high chair using arms (max reps 30sec)								
STS - normal chair w/out arms (5 reps)								
STS -normal chair w/out arms (max reps 30sec)								
STS - couch								
STS - carrying medicine ball (max reps 30sec)								

2. Sit on Floor and Stand Up

Exercise Session	1	2	3	4	5	6	7	8
Chair to aid w/ BOTH descent & ascent (time)								
Chair to aid w/ EITHER descent OR ascent (time)								
No chair (time)								

3. Alternate Stair Tapping (AT)

Exercise Session	1	2	3	4	5	6	7	8
AT - 10 reps w/ arm support (time)								
AT - 10 reps no support (time)								
AT - alternative arm lift (time)								

✓= successful, ✻ = Try Again, P = Pain, FT = fingertip, D= Dizzy

Modified from: Beauchamp M, Janaudis-Ferreira T, Parreira V et al. A randomized controlled trial of balance training during pulmonary rehabilitation for individuals with COPD. Chest 2013;144:1803-10.

GAIT EXERCISES

1. Walking in Parallel Bars

Exercise Session	1	2	3	4	5	6	7	8
Tandem walk - w/ light finger support								
Tandem walk - no support								
Tandem walk - spelling name backwards								
Sideways walk								
Backwards walk								
Low-level obstacle course - noodles, foam, block w/ support								
Low-level obstacle course - noodles, foam, block no support								
High-level obstacle course - wobble board, Bosu ball, widely spaced noodles w/ support								
High-level obstacle course - wobble board, Bosu ball, widely spaced noodles no support								

2. Walking in open space

Exercise Session	1	2	3	4	5	6	7	8
Kick a ball back & forth								
Fast walking (over 6m) with walking aid								
Fast walking (over 6m) without walking aid								
Change in speed (“fast!” “slow!”)								
Quick direction change (“change”)								
Walk and look at side visual target								
Walk and count backwards								
Walk over obstacle								
Walk, turn and sit down								
Walk, turn and sit down with cognitive task								

✓= successful, ✻ = Try Again, P = Pain, FT = fingertip, D= Dizzy

Modified from: Beauchamp M, Janaudis-Ferreira T, Parreira V et al. A randomized controlled trial of balance training during pulmonary rehabilitation for individuals with COPD. Chest 2013;144:1803-10.

STRENGTH AND RANGE OF MOTION EXERCISES

1. Lower leg

Exercise Session	1	2	3	4	5	6	7	8
Toe raises - w/ arm support (2 x 10 reps)								
Toe raises - no arm support (10 reps, 3 sec hold)								
Heel raises - w/ arm support) (2 x 10 reps)								
Heel raises -no arm support (10 reps,3 sec hold)								
Walk on toes								
Walk on heels								

2. Upper leg

Exercise Session	1	2	3	4	5	6	7	8
Step-ups - w/ arm support (2 x 10 reps)								
Step-ups - no arm support (2 x 10 reps)								
Lateral step ups - w/arm support (2 x 10 reps)								
Lateral step ups - no arm support (2 x 10 reps)								
Side stepping - w/ theraband (2 x 10 steps)								

3. Core strengthening on ball

Exercise Session	1	2	3	4	5	6	7	8
Sit on chair - weight shift, eyes closed								
Sit on cushion - weight shift								
Sit on cushion - weight shift, eyes closed								
Sit on ball - hold 1 min								
Sit on ball - weight shift								
Sit on ball - tap toes								
Sit on ball - knee raise								
Sit on ball - straighten knee								
Sit on ball - arm lifts								
Sit on ball - move arms and legs								

✓= successful, ✻ = Try Again, P = Pain, FT = fingertip, D= Dizzy

Modified from: Beauchamp M, Janaudis-Ferreira T, Parreira V et al. A randomized controlled trial of balance training during pulmonary rehabilitation for individuals with COPD. Chest 2013;144:1803-10.
