# Supplementary material for: Pulmonary Rehabilitation With Balance Training for Fall Reduction in Chronic Obstructive Pulmonary Disease: Protocol for a Randomized Controlled Trial
Source: JMIR Res Protoc. 2017 Nov 20;6(11):e228. doi: 10.2196/resprot.8178 (PMC5715200; doi:10.2196/resprot.8178)
Supplement: Multimedia Appendix 2 [file resprot_v6i11e228_app2.pdf]

Notice of Decision - Brooks, Dina

Program: Project Grant
Competition: 201603PJT
Application Number: 363312
Title: Randomized controlled trial of balance training for fall reduction in individuals with COPD

Applicants: Brooks, Dina

Beauchamp, Marla; Goldstein, Roger; Marques, Alda; Dechman, Gail; Moineddin, Rahim; et al

Research Institution: University of Toronto

Institution paid: University of Toronto

Competition Outcome:

Decision on your application: Approved
Approved Average Funding Amount: $902,111 **Term:** 3 **year(s)** 0 **month(s)**

Number of Applications Received at Stage 1: 3819
Number of Applications Accepted for Final Assessment Stage: 583
Number of Applications Approved: 491

Full competition results: http://www.cihr-irsc.gc.ca/e/49051.html

**Stage 1**
Decision on your application: Accepted for Stage 2
Consolidated Rank: 85.5133
Standard Deviation: 2.0919

Distribution of reviewer rankings

Percent	Number of Reviewers
90%-100%	0
80%-89%	3
70%-79%	0
60%-69%	0
50%-59%	0
0%-49%	0

CIHR IRSC

Institute of Aboriginal Peoples' Health
Institute of Aging
Institute of Cancer Research
Institute of Circulatory and Respiratory Health
Institute of Gender and Health
Institute of Genetics
Institute of Health Services and Policy Research
Institute of Human Development and Child and Youth Health
Institute of Infection and Immunity
Institute of Musculoskeletal Health and Arthritis
Institute of Neurosciences, Mental Health and Addiction
Institute of Nutrition, Metabolism and Diabetes
Institute of Population and Public Health

Institut de la santé des Autochtones
Institut du vieillissement
Institut du cancer
Institut de la santé circulatoire et respiratoire
Institut de la santé des femmes et des hommes
Institut de génétique
Institut des services et des politiques de la santé
Institut du développement et de la santé des enfants et des adolescents
Institut des maladies infectieuses et immunitaires
Institut de l'appareil locomoteur et de l'arthrite
Institut des neurosciences, de la santé mentale et des toxicomanies
Institut de la nutrition, du métabolisme et du diabète
Institut de la santé publique et des populations

July 15, 2016

Dr. Dina Brooks
Department of Physical Therapy
160-500 University Avenue
Toronto, Ontario M5G 1V7

Dear Dr. Brooks,

On behalf of the Canadian Institutes of Health Research (CIHR), I am pleased to inform you that your recent application to the Project Grant – Spring 2016 competition entitled “Randomized controlled trial of balance training for fall reduction in individuals with COPD” has been approved for funding.

Your application reviews and competition results can be accessed through ResearchNet. If you are unable to view these documents, please contact us at support@cihr-irsc.gc.ca. Your Authorization for Funding will follow in the mail.

As CIHR does not notify co-applicants of the decision, we ask that you inform those individuals involved, along with their research institutions (if different from your own) of the outcome of this application.

Should you have any questions, please do not hesitate to communicate with a Processing Officer in the Contact Centre at 613-954-1968 or by e-mail: support@cihr-irsc.gc.ca.

Congratulations on your success in this competition.

Sincerely,

Martine Lafrance

Martine Lafrance, Ph.D.
Manager, Project Grant Program/Investigator Initiated Research Branch
Research, Knowledge Translation and Ethics Portfolio

432976-201603PJT-PJT-363312-35000-DLPJT

CIHR IRSC

Institute of Aboriginal Peoples' Health
Institute of Aging
Institute of Cancer Research
Institute of Circulatory and Respiratory Health
Institute of Gender and Health
Institute of Genetics
Institute of Health Services and Policy Research
Institute of Human Development and Child and Youth Health
Institute of Infection and Immunity
Institute of Musculoskeletal Health and Arthritis
Institute of Neurosciences, Mental Health and Addiction
Institute of Nutrition, Metabolism and Diabetes
Institute of Population and Public Health

Institut de la santé des Autochtones
Institut du vieillissement
Institut du cancer
Institut de la santé circulatoire et respiratoire
Institut de la santé des femmes et des hommes
Institut de génétique
Institut des services et des politiques de la santé
Institut du développement et de la santé des enfants et des adolescents
Institut des maladies infectieuses et immunitaires
Institut de l'appareil locomoteur et de l'arthrite
Institut des neurosciences, de la santé mentale et des toxicomanies
Institut de la nutrition, du métabolisme et du diabète
Institut de la santé publique et des populations

July 15, 2016

Dr. Dina Brooks
Department of Physical Therapy
160-500 University Avenue
Toronto, Ontario M5G 1V7

Dear Dr. Brooks,

Congratulations on your success in the recent Canadian Institutes of Health Research (CIHR) Project Grant—Spring 2016 competition.

Your application was reviewed by your peers and considered to be of exceptionally high quality. You should take great pride in this achievement, particularly given the highly competitive nature of CIHR funding.

As you know, peer review is the cornerstone of our research funding system. This process is made possible because of the volunteerism of individuals who generously gave their time to review your application. We are continuously recruiting and retaining the most accomplished innovative and creative scientists to review health research proposals. As a CIHR-funded researcher, you may be invited to serve in the peer review process for future competitions.

To highlight your achievements and to communicate the value of health research to Canadians, we encourage you to work with your institution to promote your research. To support you in this activity, CIHR has developed guidelines on public communication available at: www.cihr-irsc.gc.ca/e/30789.html.

Once again, I offer you my congratulations and best wishes for success in your research.

Yours sincerely,

Alain Beaudet, MD, Ph.D.
President

433108-201603PJT-PJT-363312-35000-CLPJT

President

Canadian Institutes of Health Research
Room 97, 160 Elgin Street, Address locator: 4809A
Ottawa, (Ontario) K1A 0W9 Tel.: (613) 941-2672
Fax (613) 954-1800 **www.cihr-irsc.gc.ca**

Président

Instituts de recherche en santé du Canada
Pièce 97, 160 rue Elgin, Indice de l'adresse: 4809A
Ottawa, (Ontario) K1A 0W9 Tél.: (613) 941-2672
Fax (613) 954-1800 **www.irsc-cihr.gc.ca**

Canada

**Review Type / Type d'évaluation:**	Reviewer 1 / Évaluateur 1
**Name of Applicant / Nom du chercheur:**	Brooks,Dina
**Application No. / Numéro de demande:**	363312
**Agency / Agence:**	CIHR/IRSC
**Competition / Concours:**	Project Grant/Subvention Projet
**Committee / Comité:**	Project Grant Competition/Concours de subventions Projet
**Title / Titre:**	Randomized controlled trial of balance training for fall reduction in individuals with COPD

Concept/Concept

Criterion/Critère: Quality of the Idea/Qualité de l'idée

Rating/Cote: E+

Strengths/Forces: This study addresses an important health problem in patients with COPD and aims to determine if balance training can help reduce falls in this vulnerable group. The rationale for this intervention is well presented and is substantiated by the investigators' previous work which determined that this approach to intervention improves balance in this population. This study is the next logical step as falls are are associated with significant morbidity and mortality and reducing falls is the important outcome of interest. The use of an RCT to address this research question is the most appropriate study design to respond to this question.

Weaknesses/Faiblesses: It would be a useful addition to this study to investigate the relationship between the secondary outcome measures (balance, balance confidence and strength) and falls in order to better understand which of these factors may be related to an increased risk of falls.

Criterion/Critère: Importance of the Idea/Importance de l'idée

Rating/Cote: E

Strengths/Forces: A review of previous research (both from the literature and from the investigators' earlier work) strongly supports the importance of this study as a potential approach to reducing falls and associated morbidity as well as decreasing the use of health care resources in the COPD population.

Weaknesses/Faiblesses: The authors cite their previous work showing that there is a unique profile of balance impairments in individuals with COPD. It is not clear whether this profile is very different from other types of balance deficits and how this is addressed in the tailored intervention. Also, the secondary objectives of this study focuses on only one of the specific deficits (strength). To more completely understand the changes in risk of falls (primary objective of the study), it would be informative to examine some of the deficits in other areas (range of motion, posture, change in body positions and gait) as well.

Feasibility/Faisabilité

Criterion/Critère: Approach/Approche

Rating/Cote: G

Strengths/Forces: This multi-center randomized clinical trial is clearly presented, includes blinding of outcome evaluators and maintains comparable interventions between experimental and control groups. Outcome measures are standardized and include a range of outcomes.

Weaknesses/Faiblesses: One small detail- The baseline assessment should not be described as an outcome evaluation (Subjects will be required to attend a pre-rehabilitation (baseline) outcome assessment session).

The balance training intervention should be described in greater detail to provide information on how it is tailored to the needs of patients with COPD and also how it will be individualized according to each participant's needs. In addition, it is not clear how the two interventions are matched- will the balance training be added to the PR program (providing additional intervention time to the experimental group) or will the total intervention time be the same for both groups?

It is important to clarify who will be making the telephone calls to the subjects who do not return calendars and those with unclear data (I assume they must not be blinded as they will be discussing the home program to ensure proper technique and progression) and who will be making the monthly calls (will they be blinded?).

The economic evaluation is an important component of this study, however there is no mention of this aspect of the analysis in the Background or Objectives of the study.

The proposal does not discuss the potential challenges/limitations and there is no mention of strategies that will be implemented to mitigate these difficulties.

Criterion/Critère: Expertise, Experience and Resources/Expertise, expérience et ressources

Rating/Cote: E+

Strengths/Forces: Multi-disciplinary and multi-national team with complimentary expertise and previous collaborations. The team members have expertise in the area of study and this project builds upon earlier work.

Weaknesses/Faiblesses: There is no mention of how the team will provide training to the multiple sites to ensure consistency in the provision of the interventions and the outcome assessments.

**Review Type / Type d'évaluation:**	Reviewer 2 / Évaluateur 2
**Name of Applicant / Nom du chercheur:**	Brooks,Dina
**Application No. / Numéro de demande:**	363312
**Agency / Agence:**	CIHR/IRSC
**Competition / Concours:**	Project Grant/Subvention Projet
**Committee / Comité:**	Project Grant Competition/Concours de subventions Projet
**Title / Titre:**	Randomized controlled trial of balance training for fall reduction in individuals with COPD

Concept/Concept

Criterion/Critère: Quality of the Idea/Qualité de l'idée

Rating/Cote: E++

Strengths/Forces: The idea is creative and has a strong rationale. This group has demonstrated that the surrogate outcome of balance can be improved with a balance training program as part of PR, and is now seeking to extend this argument by demonstrating effect on falls and cost-effectiveness. If positive, this study will be highly impactful as it will imply a benefit for balance training as part of all future PR for COPD patients at risk for falls. If cost effectiveness is proven, this can be widely adopted. Goals and objectives are clear.

Weaknesses/Faiblesses: None.

Criterion/Critère: Importance of the Idea/Importance de l'idée

Rating/Cote: E++

Strengths/Forces: The idea in important in that a positive study will result in heath knowledge which is likely to lead to a broad change in practice, with the caveat that PR itself is poorly implemented in most jurisdictions.

Weaknesses/Faiblesses: It would have been nice to hear about some of the barriers that might make it challenging to adopt this program widely, particularly the limited capacity for PR itself, and whether and how aditional time burdens for balance training will further strain a system which has insufficient capacity for general PR, and the impact of that "opportunity cost" (ie more patients getting balance training might mean less patients getting PR).

Feasibility/Faisabilité

Criterion/Critère: Approach/Approche

Rating/Cote: E

Strengths/Forces: Methods are very robust and clearly delineated. Authors are proposing an RCT with blinded outcome assessment, and have successfully conducted an RCT of the same intervention previously. The proposal for the economic analysis includes both cost-effectiveness and cost-utility analyses, and is very robust in design. Timelines seem realistic and well thought-out, again with the benefit of a previous similar RCT. Drop-out rates are accounted for and although authors do not predict any other major obstacles, this is reasonable because they have successfully conducted a similar trial before.

Weaknesses/Faiblesses: Both groups will receive monthly phone calls and 3 home visits by a physiotherapist. - cost

Authors do not discuss the fact that many patients with COPD have walking aids. These patients should be recrutied in a stratified manner to prevent group imbalance (no pun intended), and should be assessed in a subgroup analysis.

One of the main issues with PR is the issue of poor compliance with home maintenance after completion of the program, and corresponding waning of benefits. The same issue likely applies here. Autyhors should measure and report what proportion of patients maintained their exercises, and whether those that didn't lost the benefits, and if so, how quickly. This should be looked at for the entire study after the initial PR. it also would have been nice to have a 2 year follow-up (ie one year after last contact) to re-evaluate some of these benefits, because the the q3 months visits and monthly phone calls during the study may "falsely" sustain the intervention until that point, but are not realistic long term.

Cost analysis will compare costs in int and control groups, but control group will be receiving monthly calls and quarterly visits, which is an extra cost that is above and beyond standard of care currently - this cost should not be counted as standard of care.
The primary outcome is patient-reported and patients are not blinded to the intervention - would have been better to validate falls with physician/hospital records/admin databases, though admittedly difficult to do so. In this regard, it is a strength that patients are not being informed of the nature of the balance training and its goals.

Criterion/Critère: Expertise, Experience and Resources/Expertise, expérience et ressources

Rating/Cote: O+

Strengths/Forces: A very experienced group with an excellent track record, have done this kind of trial before, and have a large cohort available to them. Infrastructure, commitment, definition of roles are all excellent.

Weaknesses/Faiblesses: Also unclear if authors have successfully completed multi-center trials with other countries before, but they have stated that they have successfully collaborated with all of these centers.

**Review Type / Type d'évaluation:**	Reviewer 4 / Évaluateur 4
**Name of Applicant / Nom du chercheur:**	Brooks,Dina
**Application No. / Numéro de demande:**	363312
**Agency / Agence:**	CIHR/IRSC
**Competition / Concours:**	Project Grant/Subvention Projet
**Committee / Comité:**	Project Grant Competition/Concours de subventions Projet
**Title / Titre:**	Randomized controlled trial of balance training for fall reduction in individuals with COPD

Concept/Concept

Criterion/Critère: Quality of the Idea/Qualité de l'idée

Rating/Cote: O+

Strengths/Forces: The intervention is stated to be taylored to issues in COPD, and although I cannot evaluate this, it would be an excellent aspect of the proposal. the inclusion of the economic evaluation may greatly enhance the impact of an intervention which is found to be effective.

Weaknesses/Faiblesses: It is not normally considered adequate address sex and gender by simply acknowledging that both men and women are to be recruited.

Criterion/Critère: Importance of the Idea/Importance de l'idée

Rating/Cote: O+

Strengths/Forces: The proposal is well focussed on a clear intervention of a well specified problem in a specific clinical population, which is normally an indicator of the potential for success. It is previously shown that exercise interventions in this population have success in reducing falls, and this may also be true for balance training.

Weaknesses/Faiblesses: ..

Feasibility/Faisabilité

Criterion/Critère: Approach/Approche

Rating/Cote: O++

Strengths/Forces: Pre-test PR assessment followed by randomization improves the comparabilitiy of the groups.   The proposed design includes good control of the contact and assessment schedule to improve interpretation of the group comparisions in terms of substative features of the treatment.

monthly rates of falls will be collected, albeit retrospectively. If there are sufficient monthly falls it may be worthwhile to examine the trajectory of the treatment effect.

assumptions of the sample size calculation are well specified.

the multcentre design with adjustment for clustering is very practical. Approriate analysis for the primary count outcome is specified.

Weaknesses/Faiblesses: Primary outcome is the number of falls. Its not clear that 1.2 falls vs. 0.84 per person year is clinically large difference given the considerable intensity of the intervention.

Criterion/Critère: Expertise, Experience and Resources/Expertise, expérience et ressources

Rating/Cote: O++

Strengths/Forces: team is comprehensive and well able to conduct the research, with the required partnerships available.

biostatistics and health economic expertise is included on the team.

Weaknesses/Faiblesses: .

NOTE

Your application was assessed by the Stage 1 reviewers. Based on the ranking of your application relative to the other applications in the competition, it was identified as highly competitive and did not require further discussion by the Final Assessment Stage committee. As a result, no Scientific Officer notes were generated. The reviewer reports from Stage 1 are available on ResearchNet.

Please refer to the *Notice of Decision* for more information regarding the ranking of your application.

For information regarding the Project Grant Program peer review process, please refer to the *Peer Review Manual - Project* (http://www.cihr-irsc.gc.ca/e/49564.html).

Votre demande a été évaluée par les évaluateurs de l'Étape 1. Compte tenu du classement de votre demande par rapport aux autres demandes dans le concours, celle-ci a été identifiée comme étant très compétitive et ne nécessitant pas de discussion par le comité de l'étape d'évaluation finale. Par conséquent, il n'y a pas de notes de l'agent scientifique. Les rapports d'évaluation de l'Étape 1 sont disponibles sur RechercheNet.

Veuillez consulter l'avis de décision pour plus d'information sur le rang de votre demande.

Pour plus d'information sur le processus d'évaluation par les pairs du Programme de subventions Projet, veuillez consulter le *Guide d'évaluation par les pairs – Projet* (http://www.cihr-irsc.gc.ca/f/49564.html).

Canadian Institutes of Health Research
Rm 97, 160 Elgin St., Address Locator 4809A
Ottawa, Ontario K1A OW9 Tel (613) 941-2672
Fax: (613) 954-1800 **www.cihr.ca**

Instituts de recherche en sante du Canada
Pièce 97, 160, rue Elgin, Indice de l'adresse 4809A
Ottawa, Ontario K1A OW9 Tél (613) 941-2672
Fax : (613) 954-1800 **www.irsc.ca**
